# Erysipelas Occuring with Parturition

**Published:** 1883-02

**Authors:** Bernard Bartow


### Erysipelas Occurring with Parturition.

Reported by Bernard Bartow, M. D.

Mrs.——, æt. 27, was in the eighth month of her second pregnancy when her child, twenty months old, was taken sick with scarlatina. For two days she carried or held the child continually. From this cause she herself was prostrated, and had lumbar and abdominal pain, and frequent vomiting, compelling her to go to bed. She had been sick in bed one day when I first saw her (Dec. 23d). Her stomach had retained nothing for twenty-four hours; there was intermittent lumbar and abdominal pain, abdominal tenderness on pressure, burning pain in epigastrium; pulse 86, temperature 98.5°. The urinary secretion was normal in quantity and phosphatic. There was no constipation. She had not had scarlatina. Until the third day following, her condition did not materially change, notwithstanding the free use of morphia hypodermically, gastric sedatives, hot application to abdomen, etc. During that time the pulse and temperature continued normal. The afternoon of Dec. 25th she felt a sudden pain in the right hypochondrium; this was followed by a chill and rise of pulse and temperature—the former being 120, the latter 100.5°. The temperature quickly fell to normal and remained so, but during the 26th and 27th, the pulse did not fall below 120 in frequency, and at times beating 140 and 160 per minute. There was no corresponding acceleration of respiration. Until the latter date morphia was administered hypodermically to relieve the inclination to vomit that was constantly present. The patient then spoke of the absence of the fœtal movements, during the preceding twenty-four hours; after that morphia, hypodermically, was discontinued, and given per orem instead, the danger of inducing narcotism of the fœtus being less by the latter method.

The increased prostration present at that time, and the continued rapid pulse, led me to think there might have been partial detachment of the placenta at the time of having the pain and chill (Dec. 25th). Her condition also had a resemblance to septicæmia, except that there was no elevation of temperature. From the susceptibility of the patient to scarlatina, it was also thought possible that the disturbance was due to that poison manifesting itself in an unusual manner, from the influence of a co-existing pregnancy. Dec. 28th, Dr. M. D. Mann saw the patient with me. Since discontinuing morphia hypodermically the movements of the fœtus had become quite strong (in the meantime the patient continued taking morphia per orem); the placenta was intact; there was slight abdominal pain or pressure; respiration and temperature normal; pulse 135. The prostration of the patient was quite severe, and beef tea and brandy only could be retained in small quantities. No diagnosis was made; but Dr. Mann was of the opinion that something was in process of development that would shortly "explode" and define the case; he advised Infus. Digitalis ʒj. every third hour to lower the heart's action. The following morning the pulse had fallen to 96. The patient complained of pain in and around the right ear; the skin in those parts was puffy and tender to pressure. Dec. 30th, an erysipelatous eruption appeared on the right ear and the side of the face and neck, and during the following forty-eight hours spread over one-half of the face. The pulse was not greatly disturbed, and the temperature not all, by this event. After that the patient showed decided improvement of strength, and nourishment and stimulants in larger quantities were retained by the stomach. The third day after the appearance of erysipelas the woman was taken with labor pains. Coincidently there was an aggravation of all her other symptoms. The erysipelas spread over the entire face; parts in which the eruption had subsided again became red, swollen and painful. The temperature rose to 102°, and the pulse to 140 per minute. Labor was completed in six hours. The child did not breathe well for several minutes after birth, and then the respirations were not vigorous. Before labor was ended the patient was moved into an unoccupied room, and as far as practicable antiseptic precautions were employed. I made very few vaginal examinations, and was careful before each examination to wash my hand and arm with strongly carbolized water. Shortly before the head was expelled I gave the patient ten grains of quinine with a view to securing firm uterine contraction. Quinine in five grain doses was repeated every fourth hour. Directed brandy milk and beef tea to be given the patient as freely as could be borne; also, that carbolized cloths be kept continually over the vulva.

The day following her delivery the woman's pulse had fallen to 90, and the temperature to 99°; and until the fifth day there was no disturbance of pulse or temperature, or any unfavorable symptoms that were dependent upon parturition. The erysipelas, however, continued to spread over the face and neck, and down the back until it reached the waist. The lochia were scanty but otherwise normal. Lactation was wholly absent. The infant lived only twenty-four hours, death beginning in the respiratory centre; life was maintained artificially six hours, during which there were frequent convulsions.

The sixth day after confinement the patient had a severe chill. Following this there was a gradual rise of pulse and temperature; by the eighth day the pulse reached 120, and the temperature 104½°. Pain over the uterus and in the lower part of the abdomen, and offensive lochia accompanied this change. During the forty-eight hours following the chill she took sixty grains of quinine, which induced cinchonism, but had no effect to reduce the temperature. The evening of the 8th day the uterus was washed out with one quart 3 per cent. solution of carbolic acid. The temperature fell 2° in the following ten hours, and the pulse from 120 to 108. The uterine injection was repeated the next morning, and at intervals of ten hours, for three ensuing days. After each operation the patient's temperature would fall 2° in the two hours immediately following, then it would slowly rise to 103½°. The intra-uterine injections were used four days; all local signs of metritis disappearing, the injections were then discontinued. The temperature did not rise above 103°, and the pulse averaged about 104. She was then able to bear large doses of quinine and stimulants, and nourishment in good quantity.

At that time (the 11th day after confinement), she had a relapse of the erysipelas. The eruption again appeared on the face, and this time in a phlegmonous form; it re-appeared in a simple form on the back and gradually spread downward to the buttocks and forward over the front of the thorax. Forty-eight hours afterward the patient became delirious, had severe subsultus, high temperature, and a weak, frequent pulse. The third day of the relapse (15th day after confinement), there was extensive hypostatic congestion of the lungs. During the last two days of her illness the respirations continued at 40 per minute, often increasing to 50 and 60; the pulse varied from 140° to 180°, and the temperature from 105.5° to 106.5°. No signs of metritis or peritonitis were present during the relapse. The delirium continued to the end; death from exhaustion taking place the 24th day of her illness, and the 17th day after parturition.

